# Metabolic Diversity and Aero-Tolerance in Anammox Bacteria from Geochemically Distinct Aquifers

**DOI:** 10.1128/msystems.01255-21

**Published:** 2022-02-22

**Authors:** Olivia E. Mosley, Emilie Gios, Louise Weaver, Murray Close, Chris Daughney, Rob van der Raaij, Heather Martindale, Kim M. Handley

**Affiliations:** a School of Biological Sciences, The University of Auckland, Auckland, New Zealand; b Institute of Environmental Science and Research, Christchurch, New Zealand; c National Institute of Water and Atmospheric Research, Wellington, New Zealand; d GNS Science, Lower Hutt, New Zealand; California Department of Water Resources

**Keywords:** aquifer, groundwater, anammox, “*Candidatus* Brocadiae”, ammonia oxidizers, aero-tolerance

## Abstract

Anaerobic ammonium oxidation (anammox) is important for converting bioavailable nitrogen into dinitrogen gas, particularly in carbon-poor environments. However, the diversity and prevalence of anammox bacteria in the terrestrial subsurface—a typically oligotrophic environment—are little understood. To determine the distribution and activity of anammox bacteria across a range of aquifer lithologies and physicochemistries, we analyzed 16S rRNA genes and quantified hydrazine synthase genes and transcripts sampled from 59 groundwater wells and metagenomes and metatranscriptomes from an oxic-to-dysoxic subset. Data indicate that anammox and anammox-associated bacteria (class “*Candidatus* Brocadiae”) are prevalent in the aquifers studied, and that anammox community composition is strongly differentiated by dissolved oxygen (DO), but not ammonia/nitrite. While “*Candidatus* Brocadiae” diversity decreased with increasing DO, “*Candidatus* Brocadiae” 16S rRNA genes and hydrazine synthase (*hzsB*) genes and transcripts were detected across a wide range of bulk groundwater DO concentrations (0 to 10 mg/L). Anammox genes and transcripts correlated significantly with those involved in aerobic ammonia oxidation (*amoA*), potentially representing a major source of nitrite for anammox. Eight “*Candidatus* Brocadiae” genomes (63 to 95% complete), representing 2 uncharacterized families and 6 novel species, were reconstructed. Six genomes have genes characteristic of anammox, all for chemolithoautotrophy. Anammox and aerotolerance genes of up to four “*Candidatus* Brocadiae” genomes were transcriptionally active under oxic and dysoxic conditions, although activity was highest in dysoxic groundwater. The coexpression of *nrfAH* nitrite reductase genes by “*Candidatus* Brocadiae” suggests active regeneration of ammonia for anammox. Our findings indicate that anammox bacteria contribute to loss of fixed N across diverse anoxic-to-oxic aquifer conditions, which is likely supported by nitrite from aerobic ammonia oxidation.

**IMPORTANCE** Anammox is increasingly shown to play a major role in the aquatic nitrogen cycle and can outcompete heterotrophic denitrification in environments low in organic carbon. Given that aquifers are characteristically oligotrophic, anammox may represent a major route for the removal of fixed nitrogen in these environments, including agricultural nitrogen, a common groundwater contaminant. Our research confirms that anammox bacteria and the anammox process are prevalent in aquifers and occur across diverse lithologies (e.g., sandy gravel, sand-silt, and volcanic) and groundwater physicochemistries (e.g., various oxygen, carbon, nitrate, and ammonium concentrations). Results reveal niche differentiation among anammox bacteria largely driven by groundwater oxygen contents and provide evidence that anammox is supported by proximity to oxic niches and handoffs from aerobic ammonia oxidizers. We further show that this process, while anaerobic, is active in groundwater characterized as oxic, likely due to the availability of anoxic niches.

## INTRODUCTION

For decades, loss of fixed nitrogen (N) from freshwater environments, including aquifers was exclusively attributed to heterotrophic denitrification (1). Ammonium (NH_4_^+^) was deemed inert under anoxic conditions (2), and denitrification was considered the dominant anoxic sink for inorganic nitrogen (1). This view was altered when anaerobic ammonium oxidation (anammox), mediated by autotrophic bacteria from a deep-branching class within the phylum *Planctomycetes*, was discovered (3). Currently, only 10 anammox species have been enriched in culture (4); none are axenic. Their physiological characteristics and niches are defined using Monod model parameters (5). Anammox bacteria oxidize ammonium with nitrite (NO_2_^−^) to N_2_ gas without nitrous oxide emissions (6) and are considered chemoautotrophic (7). Therefore, they tend to outcompete heterotrophic denitrifiers in low-carbon systems (8). Here, we explored the significance of anammox in aquifers. As typically oligotrophic environments (9), aquifers may represent ideal ecosystems for chemolithoautotrophic anammox bacteria.

A growing body of research now shows that anammox bacteria play an important role in the global aquatic N cycle, based on their discovery in diverse aquatic environments, including oceanic oxygen-minimum zones (10, 11), meromictic lakes (12), marine subsurface sediments (13), uncontaminated suboxic groundwater (8), and contaminated groundwaters (14–16). Of these, aquifers harbor the largest freshwater store on Earth and can be characterized by long groundwater residence times (17), suitable for slow-growing anammox bacteria (with doubling times of 7 to 22 days) (18). Moreover, while groundwater often contains low levels of naturally occurring N species, such as nitrate (<0.25 mg/L) (19) and ammonia (<0.2 mg/L) (20), and frequent inputs of N from agricultural sources (21), aquifers tend to contain little organic carbon (9), potentially limiting heterotrophic denitrification (1) and favoring anammox bacteria. Anammox bacteria may also be able to compete with denitrifiers for organic carbon. Enrichment experiments have demonstrated that some anammox bacteria have the ability to oxidize small organic acids, such as propionate and acetate, with NO_3_^−^/NO_2_^−^ (22), pointing to greater metabolic versatility than previously assumed for these chemolithoautotrophic organisms.

Anammox relies on the presence of both oxidized and reduced inorganic N compounds and, subsequently, is highly active in redox transition zones, where N species fluctuate between oxidation and reduction reactions (23). Anammox bacteria are therefore found naturally occurring at the interface of aerobic-anaerobic conditions, where NO_2_^−^-containing water and anaerobic water with NH_4_^+^ mix (24). N fluctuation frequently occurs in groundwater due to interactions with surface water or to oxygen penetration from above in unconfined shallow aquifers (25). Elevated groundwater tables, which promote oxygenation through interactions with the overlying soil and groundwater recharge, stimulate a variety of anammox bacteria (16). Surface water contaminated with nitrogen species can be a source of NH_4_^+^ in aquifers. This ammonium can be partially absorbed by clay minerals (26) or oxidized to NO_2_^−^ or NO_3_^−^ by nitrification (1). NO_2_^−^ produced by aerobic ammonia oxidizers can serve as an electron acceptor in anammox (27). While anammox bacteria have been observed previously in groundwaters (8), their prevalence and genetic diversity are unclear. It is also not known how they are impacted by the heterogeneous chemical conditions found in aquifers.

To provide insights into anammox ecology and activity across a diverse range of groundwater ecosystems, we collected groundwater from 59 sites over 4 geographic regions across New Zealand. To the best of our knowledge, this is the largest survey of anammox communities in groundwater to date, encompassing a range of characteristics, including variations in nitrogen species and in organic carbon and dissolved oxygen concentrations. By reconstruction and metabolic characterization of diverse novel “*Candidatus* Brocadiae” genomes, and by quantifying the distribution, abundance, and activity of anammox bacteria across distinct groundwater conditions, we reveal the diverse ecological niches of these bacteria in groundwater. Results provide evidence in support of anammox as an important biological process in aquifers and a major N sink (16).

## RESULTS AND DISCUSSION

### Prevalence and activity of anammox in chemically diverse groundwaters. (i) Anammox bacteria are widespread in groundwater and phylogenetically diverse.

**FIG 1:**
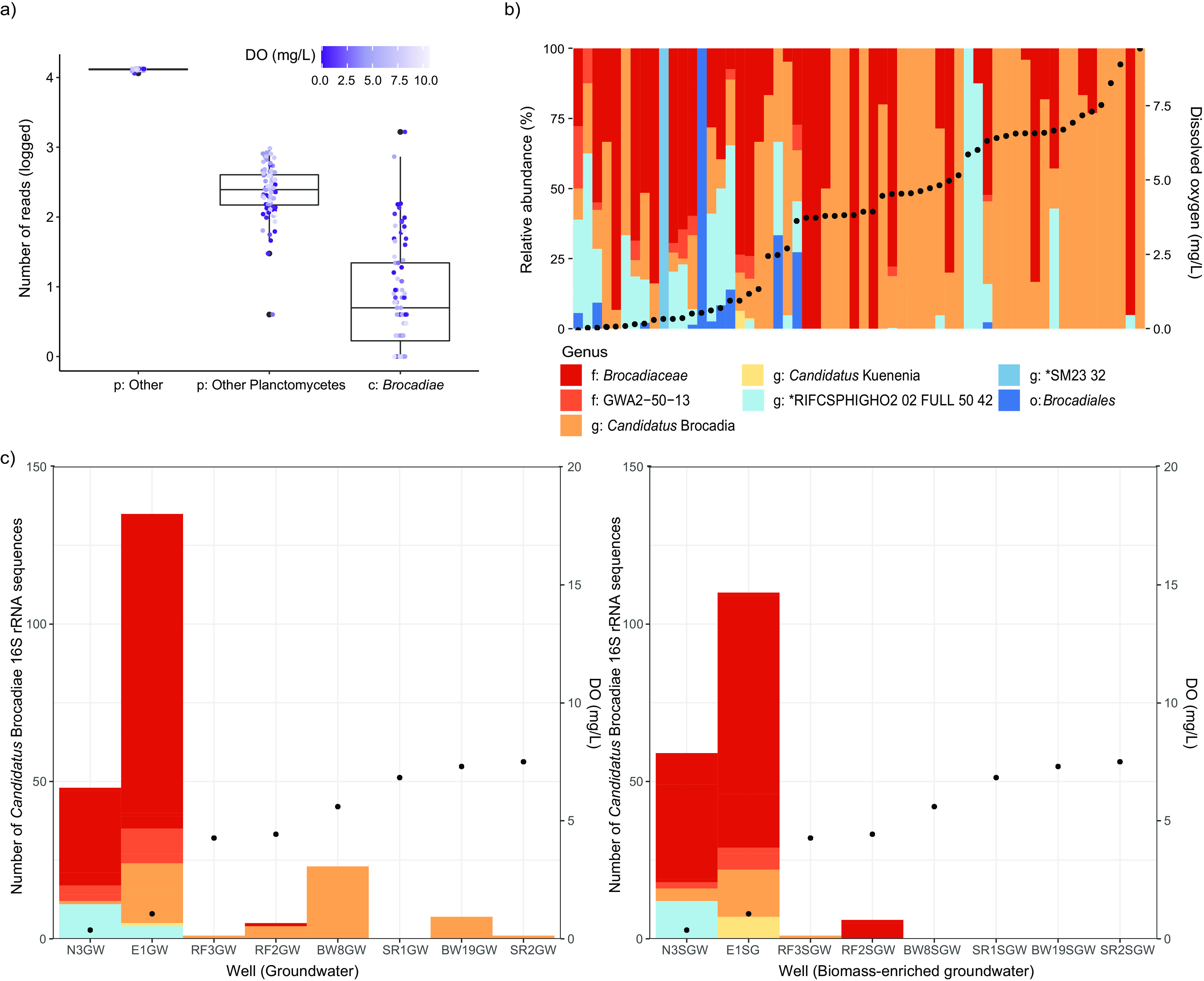
Box plot and stacked bar graphs showing the prevalence, relative abundance, and diversity of anammox bacteria (“*Ca.* Brocadiae”), based on 16S rRNA gene amplicon data. (a) Number of OTUs identified as “*Ca.* Brocadiae” relative to other bacteria (p:Other) and non-anammox *Planctomycetes* (p:Other Planctomycetes) across 80 samples. The horizontal line represents the median number of OTUs present. (b) Structure of the anammox bacterial community observed in 60 samples where “*Ca.* Brocadiae” were identified, ordered by DO concentration. (c) Structure of the anammox bacterial community observed in groundwater (left) and biomass-enriched groundwater (right), ordered by groundwater DO concentration. Black points show DO content. Bars represent different genera within the class “*Ca.* Brocadiae.” g, genus; f, family; o, order; c, class; p, phylum.

All known anammox bacteria associate exclusively with order “*Candidatus* Brocadiales” (28) in the class “*Ca.* Brocadiae.” Based on 16S rRNA gene amplicon analysis of 80 groundwater samples collected across 10 aquifers, “*Ca.* Brocadiae” was present in 60 samples (from 47 wells) across all four regions assessed in this study. Results demonstrate “*Ca.* Brocadiae” prevalence within aquifers comprising unconsolidated materials (sandy gravel = 55/71; sand/silt = 1/1), which are a common aquifer type globally (29), and consolidated volcanic rock (basalt/ignimbrite = 4/7) (Fig. 1a; Table S1). Furthermore, “*Ca.* Brocadiae” was present in groundwaters encompassing a range of nitrate N (0–22 g/m^3^), dissolved oxygen (DO) (0 to 10.6 mg/L), phosphate (0 to 16.4 g/m^3^), and dissolved organic carbon (DOC, 0 to 26 mg/L) concentrations (based on bulk groundwater measurements) (Table S1).

10.1128/msystems.01255-21.6 TABLE S1 Sample details, including well location, groundwater geochemistry, observed “*Ca.* Brocadiae” presence/absence, and ratios of *hzsB* and *amoA* (archaeal and bacterial) genes and transcripts quantified by ddPCR. BD, below detection limit. Download Table S1, XLSX file, 0.1 MB. Copyright © 2022 Mosley et al. 2022 Mosley et al. https://creativecommons.org/licenses/by/4.0/ This content is distributed under the terms of the Creative Commons Attribution 4.0 International license.

The wide range of groundwater DO concentrations across which members of the class “*Ca.* Brocadiae” were detected is consistent with findings from two pristine carbonate-rock aquifers in Germany (8). There, anammox was presumed to outcompete denitrification due to low organic carbon and contributed to 83% of total N loss. Overall, “*Ca.* Brocadiae” represented 0.37% of groundwater prokaryotic communities (3,940/1,071,440 sequences; ∼49.3 sequences ± 202 [standard deviation {SD}] per sample) and ranked 36th in abundance out of all 205 classes of bacteria and archaea. Research has shown that anammox is globally common in soils overlying aquifers, periodically saturated by high groundwater tables (16). Results here demonstrate that anammox-related bacteria are also common in the sediments and rock constituting aquifer saturated zones.

In terms of composition, the class “*Ca.* Brocadiae” comprised 37 operational taxonomic units (OTUs) (or 58 amplicon sequence variants), of which 42% were assigned to four uncultivated or enrichment-cultivated genera (Fig. S1): “*Candidatus* Brocadia” (6 OTUs), “*Candidatus* Kuenenia” (1 OTU), *Planctomycetes* bacterium RIFCSPHIGHO2_02_FULL_50_42 (6 OTUs), and *Planctomycetes* bacterium SM23_32 (3 OTUs). Over half (56.8%) could be classified only at the family (“*Candidatus* Brocadiaceae,” 10 OTUs; GWA2-50-13, 9 OTUs) and order (“*Candidatus* Brocadiales,” 2 OTUs) levels, highlighting a large degree of novelty among groundwater anammox candidates. Three of these taxonomic groups were strikingly abundant and prevalent, including the 7 most abundant OTUs overall, and representing >90% of all anammox sequences: “*Ca.* Brocadia,” RIFCSPHIGHO2_02_FULL_50_42, and those classified only as “*Ca.* Brocadiaceae” (Fig. 1b). Together with previous studies, results show that the genus “*Ca.* Brocadia” is common in terrestrial subsurface environments (8), dominating groundwater anammox communities by up to 80% (30, 31). They were present in 83% of our samples and accounted for 25% of 16S rRNA gene sequences from anammox candidates. The wide distribution of “*Ca.* Brocadia” across varied groundwater conditions, as determined here, may be attributed to its growth properties (inferred from Monod models) (5). “*Ca*. Brocadia” species exhibited the highest maximum specific growth rate among anammox bacteria, and they outcompete other freshwater anammox bacteria (5).

10.1128/msystems.01255-21.1 FIG S1 Maximum-likelihood phylogenetic tree showing 16S rRNA gene sequences classified as the class “*Ca.* Brocadiae.” Sequences recovered from groundwater in this study (bold font) comprise 16S rRNA gene amplicon OTUs and (near) full-length 16S rRNA gene sequences reconstructed from metagenomic data using EMIRGE or SPAdes with identification by Metaxa2. Reference sequences were obtained from the SILVA SSU (r183.1) database. The tree was built using the TIM3+F + I+G4 model of substitution using 1,000 bootstrap replicates and annotated in iTOL. The scale bar represents the number of substitutions per site. Colored tiles distinguish different anammox clades. Download FIG S1, TIF file, 1.8 MB. Copyright © 2022 Mosley et al. 2022 Mosley et al. https://creativecommons.org/licenses/by/4.0/ This content is distributed under the terms of the Creative Commons Attribution 4.0 International license.

### (ii) “*Ca*. Brocadiae” distribution or relative abundances were greater in suspended than attached aquifer fractions.

**FIG 2:**
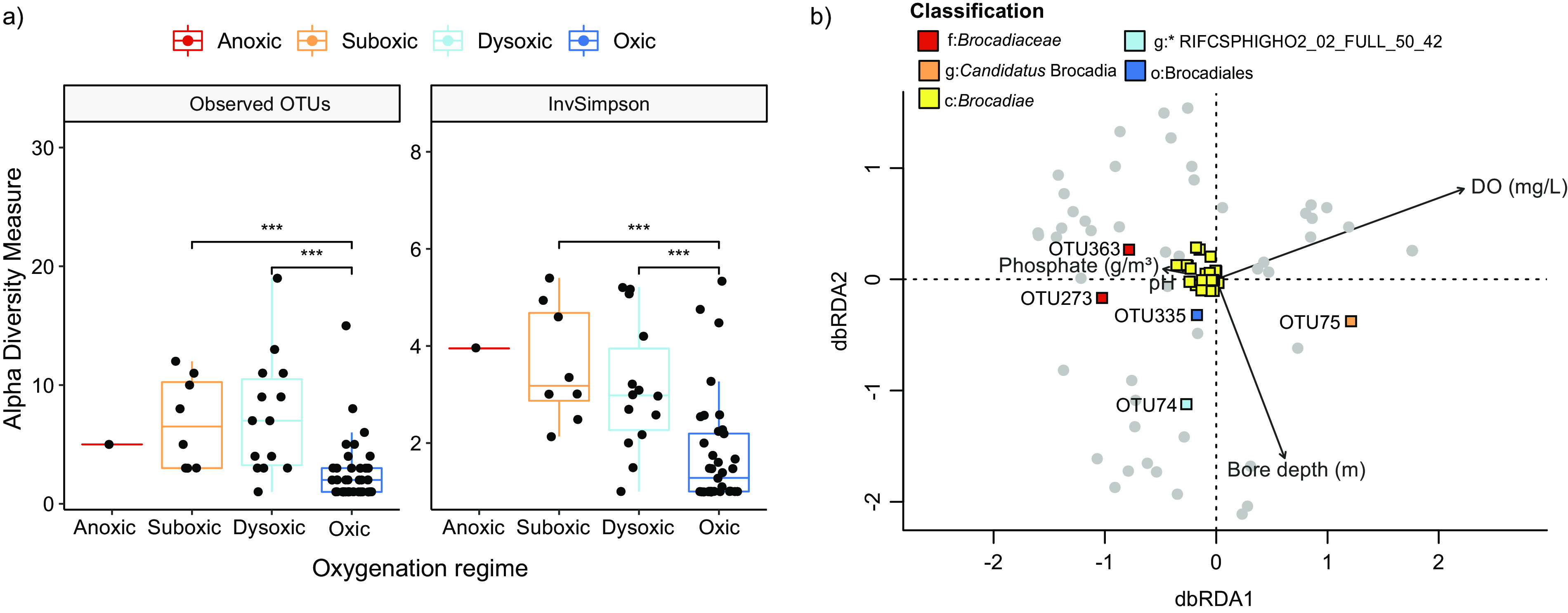
Diversity analysis and an ordination plot showing the environmental variables and factors influencing the structure of the anammox community. (a) Box plots represent observed OTUs (richness) and inverse Simpson diversity at anoxic, suboxic, dysoxic, and oxic sites (***, *P < *0.01; Wilcoxon test). (b) Distance-based redundancy analysis of the 16S amplicon data from the 60 groundwater samples using a Bray-Curtis dissimilarity matrix between samples based on OTU abundance. Vectors show significant environmental variables (excluding ORP due to missing values), constraining the variability in community composition (*P < *0.05). “*Ca.* Brocadiae” OTUs were added to the ordination using species scores (colored squares), and the 5 OTUs shown are identified to the lowest level of taxonomy (g, genus; f, family; o, order; c, class; p, phylum). Several amplicon sequence variants (ASVs) matched each of the 5 OTUs shown with 99 to 100% identity; OTU75 (9 ASVs), OTU74 (6 ASVs), OTU335 (5 ASVs), OTU363 (6 ASVs), and OTU273 (9 ASVs).

Microbial community compositions can vary considerably between suspended or planktonic biomass in groundwater and biomass attached as biofilms on aquifer sediments and rocks (32). When comparing groundwater (the planktonic fraction) and biomass-enriched groundwater postsonication (the planktonic + biofilm fraction), we found that class “*Ca.* Brocadiae” 16S rRNA gene amplicon sequences were recovered from almost all groundwater samples (seven of eight wells sampled for both fractions) but only half postsonication (Fig. 1c; Table S1). This difference is mostly explained by higher “*Ca.* Brocadiae” relative abundances in groundwater communities in the planktonic versus biofilm fractions at higher DO concentrations. Thus, “*Ca.* Brocadiae” represented a greater fraction of subsurface communities in groundwater than attached to aquifer materials. However, we identified no significant difference between these sample types (planktonic and biofilm) in terms of “*Ca.* Brocadiae” composition based on linear discriminant analysis effect size (LEfSe) analysis (Kruskal-Wallis test, *P > *0.05). Instead, oxygen was associated with a greater effect overall, as sites with lower DO concentrations contained greater “*Ca.* Brocadiae” diversity in both sample types, along with a substantially greater number of “*Ca.* Brocadiae” amplicon sequences (Fig. 1b and c and 2a).

### (iii) “*Ca*. Brocadiae” relative abundance and community composition vary strongly with dissolved oxygen.

DO concentrations ranged from oxic to anoxic and are characterized here as anoxic (0 mg/L), suboxic (<0.3 mg/L), dysoxic (0.3 to 3 mg/L), and oxic (>3 mg/L) (33). They, along with other geochemical parameters, were found to be generally stable through time in a subset of eight groundwater wells that were sampled twice, 3 to 4 months apart (Table S1). DO was positively correlated with oxidation-reduction potential (ORP), sulfate, and nitrate N and negatively correlated with ammoniacal N (detected in only 24/80 samples), phosphate, dissolved reactive phosphorus (DRP), pH, temperature, and conductivity (Spearman’s rank correlations) (Table S2). While DO was not correlated with borehole depth in this study, borehole depth was positively correlated with ammoniacal N, pH, and total dissolved solids (TDS) and negatively correlated with nitrate N and ORP, reflecting an increase in reducing conditions with well depth. DOC is generally associated with lower oxygen availability in groundwater (34); however, concentrations were near or at those consistent with oligotrophic groundwater conditions and did not exhibit a significant relationship with any of the parameters, possibly reflecting rapid utilization in groundwater (Table S1) (9).

10.1128/msystems.01255-21.7 TABLE S2 Spearman’s correlations (*R*) and adjusted *P* values (*p*) between geochemical/well parameters and between gene/transcript abundances and geochemical/well parameters (correlations are shown in black font where adjusted *P* values are ≤0.05). Download Table S2, XLSX file, 0.1 MB. Copyright © 2022 Mosley et al. 2022 Mosley et al. https://creativecommons.org/licenses/by/4.0/ This content is distributed under the terms of the Creative Commons Attribution 4.0 International license.

The overall proportional abundance of class “*Ca.* Brocadiae” 16S rRNA gene sequences was significantly and negatively correlated with DO concentrations (*r* = −0.28) (Table S2), a trend also demonstrated in batch culture experiments (35) and observed in carbonate-rock aquifers (8). Significant positive correlations were instead found with DOC (*r *= 0.29), which was uncorrelated with DO, and also conductivity (*r *= 0.27), phosphate (*r *= 0.31), DRP (*r *= 0.31), and NO_2_^−^ (*r *= 0.31, although only 14% of samples had concentrations above the detection limit for NO_2_^−^). Of these, as indicated above, conductivity and phosphate/DRP concentrations negatively correlated with DO overall (*P* < 0.05), and NO_2_^−^ was also weakly negatively correlated (*P* = 0.05). Correlations between “*Ca.* Brocadiae” and other substrates, including those critical for ammonia oxidation (NH_4_^+^ and NO_3_^−^), were not significant, indicating that parameters such as DO, nitrite, and DOC are more important factors for determining the presence of anammox bacteria. In addition to these parameters, multivariate regression tree analysis showed that redox potential (ORP threshold, 175.9 mV) was the main factor discriminating “*Ca.* Brocadiae” communities (Fig. S2).

10.1128/msystems.01255-21.2 FIG S2 Multivariate regression tree analysis generated using mvpart v1.6.2 (https://cran.r-project.org/src/contrib/Archive/mvpart/) of the relation between abundance of anammox (grouped at lowest level of identification, from the order “*Ca.* Brocadiales” to genus) and environmental parameters in groundwater samples. ORP values are in millivolts, DO is in milligrams per liter, SPC is in microsiemens per centimeter, and nitrate N and DOC are in grams per cubic meter. GW, groundwater; SGW, sonicated groundwater. *, *Planctomycetes* bacterium. Bar plots show the 16S rRNA gene-based relative abundance of anammox bacteria shown in the key. Download FIG S2, EPS file, 1.3 MB. Copyright © 2022 Mosley et al. 2022 Mosley et al. https://creativecommons.org/licenses/by/4.0/ This content is distributed under the terms of the Creative Commons Attribution 4.0 International license.

As indicated by comparisons of planktonic and attached “*Ca.* Brocadiae” fractions (Fig. 1c), analysis of anammox community diversity by oxygenation regime (anoxic, suboxic, dysoxic, and oxic) across all samples revealed that OTU richness and alpha diversity (inverse Simpson) were significantly higher at the suboxic and dysoxic sites than oxic sites (Fig. 2a), suggesting that groundwater with low oxygen supports more anammox species. The spatial distribution of different “*Ca.* Brocadiae” taxa was significantly associated with changes in DO and borehole depth (distance-based redundancy analysis [dbRDA]) (Fig. 2b), along with aquifer location and lithology, phosphate, and pH, which collectively explained 16% (*R*^2^ adjusted) of the variation (permutation test; *P* < 0.05, permutations = 999). When only DO was considered, dramatic differences in composition were evident (Fig. 1b). Notably, the relative abundance of the genus “*Ca.* Brocadia” fraction was positively correlated with DO concentrations (*r *= 0.46) (Fig. 1b). Three “*Ca.* Brocadia” and “*Ca.* Brocadiaceae” OTUs made up 30.4 to 89.0% of the anammox-associated community in groundwater with >4 mg/L DO (Fig. S3-S4).

10.1128/msystems.01255-21.3 FIG S3 (a) A random fit tree generated using the APE package (RStudio) and plotted with phyloseq using 16S rRNA OTUs of class “*Ca.* Brocadiae” from amplicon data showing presence/absence at sites. Points represent sites and are colored according to oxygen regimen (anoxic, uboxic, dysoxic, and oxic). Random branch lengths were generated using runif. The 7 most abundant OTUs are in bold. (b) Cluster dendrogram showing hierarchical cluster analysis on the Bray-Curtis dissimilarity between groundwater samples labeled with oxygen regimen and clustered with the ward.D2 method. Download FIG S3, EPS file, 2.5 MB. Copyright © 2022 Mosley et al. 2022 Mosley et al. https://creativecommons.org/licenses/by/4.0/ This content is distributed under the terms of the Creative Commons Attribution 4.0 International license.

Oxygen tolerance in anammox bacteria has been demonstrated previously (36, 37), particularly for “*Ca.* Brocadia.” For example, “*Ca.* Brocadia sinica” exhibited tolerance in the presence of >1 mg/L DO *in vitro* (38), and anammox bacteria closely related to “*Ca.* Brocadia fulgida” have been reported in groundwater with up to 3 mg/L oxygen (8) (>3 mg/L DO is generally classified as oxic [33]). A recent study analyzing the metabolism of “*Ca.* Brocadia” species identified genes encoding superoxide dismutase and cytochrome *c* peroxidase genes that are involved in oxygen detoxification (39), as might be expected for organisms living at reduction-oxidation interfaces. “*Ca.* Brocadiae” overall were detected in groundwater with much higher DO concentrations in this study (up to 10.6 mg/L), and results indicate that “*Ca.* Brocadia” taxa are better adapted to higher-oxygen conditions. However, as aquifers are spatially heterogenous and anammox bacteria require access to reduced forms of nitrogen, it is likely that “*Ca.* Brocadiae” inhabit relatively lower-oxygen niches within the sampled aquifers than is reflected by bulk groundwater measurements. As such, the upper limits of DO tolerance for anammox bacteria require further validation. In comparison, a further 25.9% of “*Ca.* Brocadiae” comprised *Planctomycetes* RIFCSPHIGHO2_02_FULL_50_42 (mostly OTU 74). The overall relative abundance was significantly and negatively correlated with DO (*r =* −0.38) and ORP (*r =* −0.45), indicating a preference for reducing conditions (*P < *0.05) (Table S2). It was originally recovered from an aquifer adjacent to the Colorado River (Rifle, CO, USA) (40), with conditions similar to site Wel13 here, where the genus was most abundant, including identical dysoxic conditions (0.78 mg/L DO) with low NO_2_^−^ and NO_3_^−^.

### (iv) DOC did not negatively impact “*Ca.* Brocadiae” community abundance.

Anammox generally occurs at low organic carbon levels (41). As reported above, DOC and “*Ca.* Brocadiae” community abundances were instead positively correlated. The DOC composition was not analyzed in this study, which makes further comparisons between sites difficult. However, given the importance of redox conditions (and hence DO) for the anammox process, the observed association may be best explained by the negative relationship typically shared by DO and DOC in groundwater, where organic carbon availability regulates aerobic metabolism (34) and is associated with anoxia (although DO and DOC were not significantly correlated in this study [*r* = −0.07, *P = *0.66]) (Table S2). Nevertheless, some anammox bacteria (e.g., “*Candidatus* Anammoxoglobus propionicus”) have a higher affinity for NO_2_^−^ in the presence of organic acids, such as propionate, or can assimilate formate via the Wood-Ljungdahl pathway (as recently shown for “*Candidatus* Kuenenia stuttgartiensis”) (42), implying that groundwater DOC could support the growth of some anammox bacteria.

### (v) Hydrazine synthase capacity, but not activity, was correlated with DO.

**FIG 3:**
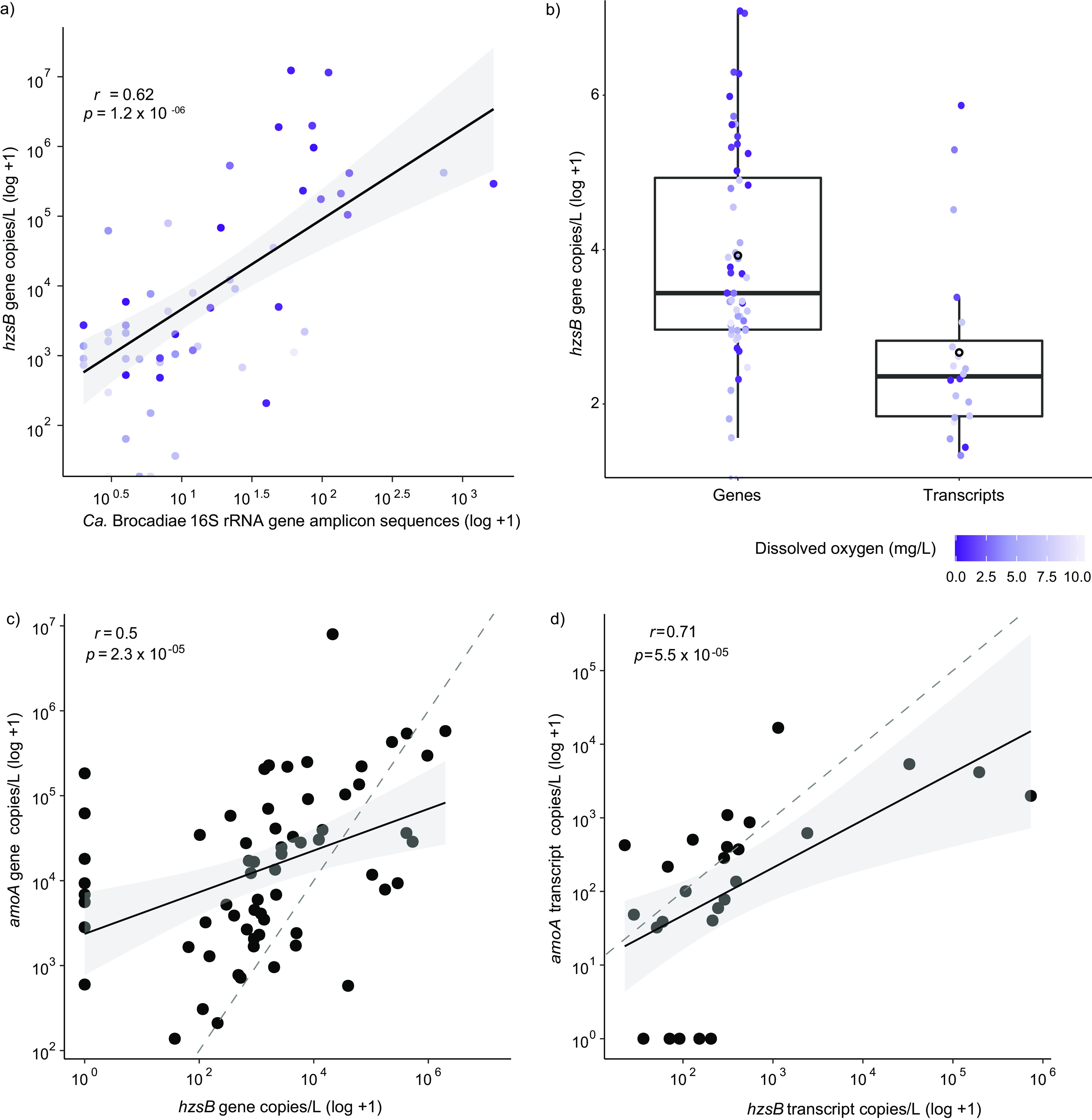
Correlations between 16S rRNA gene amplicon data, aerobic ammonia oxidizers (archaea and bacteria), and abundance of hydrazine synthase genes and transcripts from ddPCR data. (a) Correlation of *hzsB* gene copies per liter of groundwater (log_10_) and number of “*Ca.* Brocadiae” (log_10_) gene amplicon sequences from the rarefied OTU table with samples containing the class “*Ca.* Brocadiae,” showing strong agreement between quantitative data for the *hzsB* anammox functional marker gene and “*Ca.* Brocadiae” relative abundances. (b) Box plot representing log_10_ abundance of *hzsB* genes for genes and transcripts. Black open circles represent means and horizontal lines represent the medians of gene copies per liter. Purple gradients for panels a and b correspond to DO concentration. (c) Correlation of *amoA* genes (log_10_) and *hzsB* genes (log_10_) copies per liter of groundwater using Spearman’s correlation for genetic potential (DNA). (d) Correlation of *amoA* genes (log_10_) and *hzsB* genes (log_10_) copies per liter of groundwater using Spearman’s correlation in transcripts (RNA). The gray dashed line shows the theoretical 1:1 ratio.

To confirm anammox potential and activity in groundwater, we quantified genes and transcripts encoding hydrazine synthase subunit *hzsB*. Together, genes *hzsA*, *hzsB*, and *hzsC* encode hydrazine synthase, which is a key enzyme in anammox metabolism, converting nitric oxide and ammonium into hydrazine (6). The relative abundance of “*Ca.* Brocadiae” 16S rRNA gene sequences correlated strongly with *hzsB* gene copies (*r *= 0.62, *P < *0.05) (Fig. 3a), consistent with the expectation that “*Ca.* Brocadiae” undertake anammox (43). The concentration of *hzsB* genes ranged from 3.64 × 10^1^ to 1.23 × 10^7^ genes/L of groundwater (excluding 11.1% of samples below detection) with an average of 4.9 × 10^5^ ± 2.1 × 10^6^ (SD) genes/L. This is consistent with concentrations of ∼1.5 × 10^4^ to ∼1.0 × 10^8^ genes/L found elsewhere in the subsurface, including in soil horizons interacting with the water table (8, 44). The average number of *hzsB* transcript copies was significantly lower than that of *hzsB* gene copies (Wilcoxon test, *P* < 0.01; 4.5 × 10^5^ copies/L fewer, ∼13.2 times lower) (Fig. 3b), suggesting a latent capacity of 92.4%. Overall, *hzsB* gene copy numbers were negatively correlated with DO (*r* = −0.42, *P < *0.05), reflecting the trend seen with “*Ca.* Brocadiae” 16S rRNA gene relative abundance and DO, and as expected for an anaerobic process. While DO is expected to decrease with borehole depth, favoring anaerobic processes such as anammox, gene copy numbers were negatively correlated with borehole depth (*r* = −0.34). This may be explained by the weak relationship observed between DO and borehole depth in this study: DO tended to decrease with depth, but 22% of wells ≤10 m deep had DO concentrations of <3 mg/L (Table S1). No significant relationship was evident between *hzsB* gene transcripts or anammox activity and oxygen availability (*r* = −0.16, *P = *0.65) or bore depth (*r *= 0.14, *P* = 0.70).

### Co-occurrence of aerobic and anaerobic ammonia oxidizers.

The nitrification-anammox process allows for elevated N removal efficiencies from NH_4_^+^ in naturally ammonium-rich or contaminated water (45), by ensuring that a portion of ammonium is converted to nitrite. Potential combined nitrification-anammox was determined by quantification of archaeal and bacterial ammonia monooxygenase genes (*amoA*). Results show that *hzsB* and archaeal and bacterial *amoA* genes (*r *= 0.5) and transcripts (*r *= 0.71) were strongly correlated, suggesting co-occurrence of aerobic and anaerobic ammonium oxidation (*P < *0.05) (Fig. 3c and d). Co-occurrence has been observed previously within laboratory-based models (46) and an aquifer system, where anammox was the dominant process (8). Together, these results suggest that anammox in aquifers relies, at least partially, on nitrite derived from aerobic ammonia oxidation. Results also indicate that anammox is more frequently the dominant process, in terms of gene expression, when a wide range of aquifers is considered (Table S1). Although aerobic ammonia monooxygenase gene copies were more abundant in 83% of samples than hydrazine synthase genes (detected across 10 aquifers), hydrazine synthase transcripts were more abundant than ammonia monooxygenase transcripts in 69% of samples (detected across nine aquifers and higher in eight).

### Phylogenomic diversity of anammox bacteria in aquifers.

**FIG 4:**
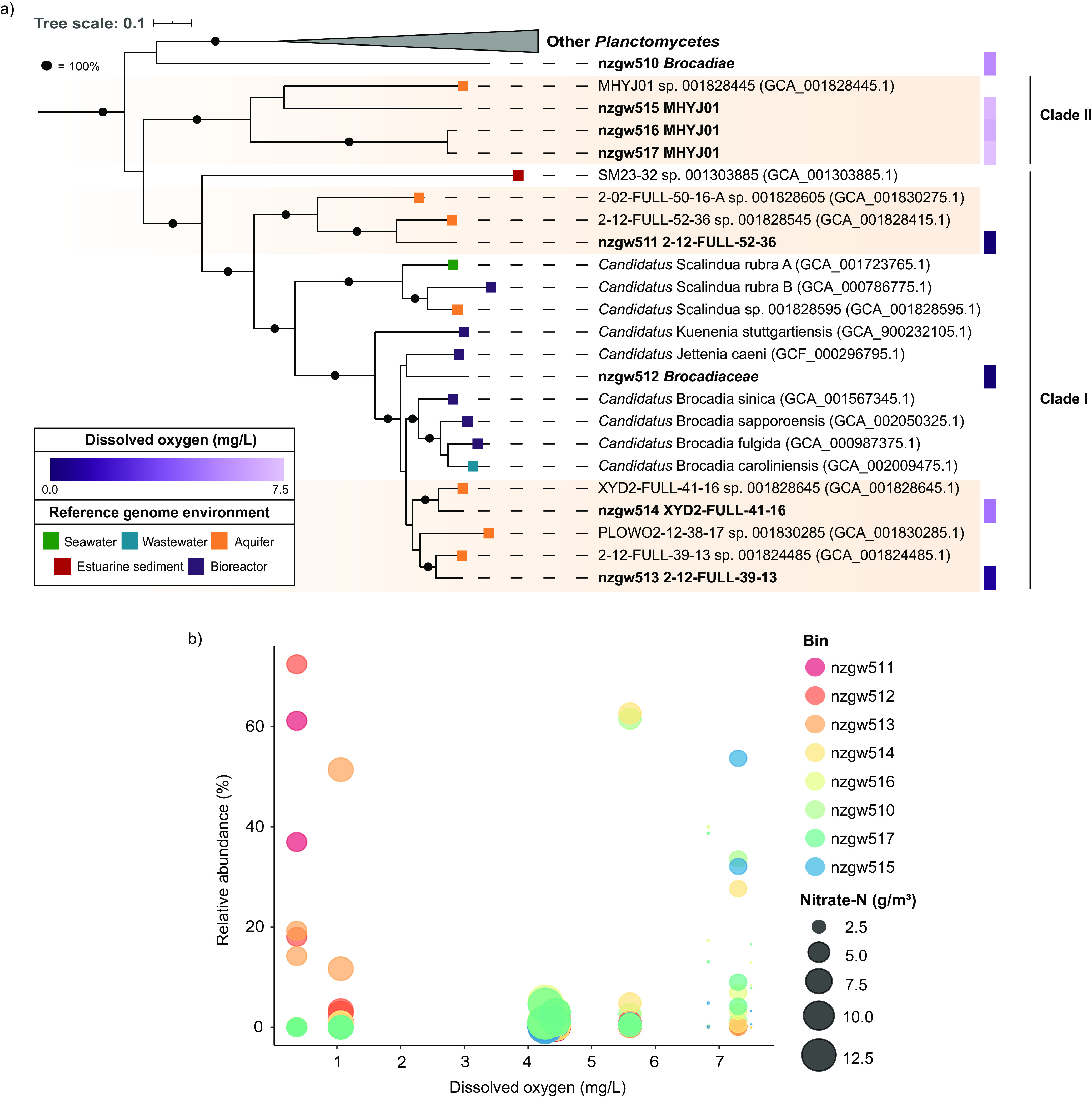
Phylogenetic distribution and relative abundance of “*Ca.* Brocadiae” MAGs. (a) Maximum-likelihood phylogenomic tree of 28 *Planctomycetes* genomes based on 120 concatenated bacterial marker genes (GTDB-Tk) (68 to 114 genes present) with 5,040 amino acid sites using the LG+F+R5 model of substitution and 1,000 bootstraps. The purple gradient represents the DO concentration at the site with highest relative genome abundance for that genome. Colored tiles represent the environment of recovery for the reference genomes. Bootstrap values shown as black circles equal 100%. The scale bar indicates the number of substitutions per site. Sequences from this study are shown in bold font, with both the study identifier and GTDB classification given. Orange shading represents clades of “*Ca.* Brocadiae” genomes recently recovered from aquifers. See Table S3 for reference genome details. (b) “*Ca.* Brocadiae” genome relative abundance across DO concentration at each site (relative to other “*Ca.* Brocadiae”). Bubble size corresponds to nitrate concentration at each site.

Genomes were reconstructed from 16 samples (gwj01 to gwj16) across eight wells (groundwater with or without biomass enrichment using sonication to detach biofilms from the surrounding aquifer materials) spanning a range of oxygen (0.37–7.5 mg/L) and nitrate (0.47–12.6 g/m^3^) concentrations (Table S1). Of 541 recovered metagenome-assembled genomes (MAGs) that were >50% complete with ≤5% contamination, eight were identified as “*Ca.* Brocadiae” (63.4 to 95.6% complete) (Table S3). Of these MAGs that contained partial or (near) full-length (445 to 1,496 bp) 16S rRNA gene sequences (nzgw513, nzgw516, and nzgw517), those from nzgw516 and nzgw517 aligned fully with OTU729 (272 bp long), which was present in 21/80 samples (total of 92 sequences). Both the nzgw516 and nzgw517 and OTU729 sequences had best hits to *Planctomycetes* spp., including *Planctomycetes* strain Pla86 and “*Ca.* Kuenenia stuttgartiensis,” when searched against the NCBI NR database (BLASTN; 79.54% to 82.85% identity), consistent with phylogenomic analyses showing that these genomes comprise a novel clade (clade II) (Fig. 4a). OTU729 was classified only to the domain *Bacteria* using USEARCH with the SILVA SSU Ref NR99 database and RDP Classifier, suggesting that these novel “*Ca.* Brocadiae” could be underestimated in the environment when a 16S rRNA gene-based approach is used.

10.1128/msystems.01255-21.8 TABLE S3 Details of “*Ca*. Brocadiae” MAGs recovered in this study (including relative abundance) and organism accession numbers for reference genomes used in whole-genome pairwise comparisons and phylogenetic analyses. Bold text indicates sample where genome was recovered. Download Table S3, XLSX file, 0.1 MB. Copyright © 2022 Mosley et al. 2022 Mosley et al. https://creativecommons.org/licenses/by/4.0/ This content is distributed under the terms of the Creative Commons Attribution 4.0 International license.

A pairwise comparison with 16 other “*Ca.* Brocadiae” genomes from various environments yielded average nucleotide identity (ANI) values below the proposed species cutoff of 95% (47) (Fig. S5), implying that the newly recovered MAGs represent novel species. The phylogenomic tree (Fig. 4) revealed that six NZ aquifer-derived MAGs form three (sub)clades with recently recovered genomes from the aquifer in Rifle, CO (40), suggesting that these are groundwater-adapted species. One (nzgw512) clustered with the well-characterized anammox bacterium “*Candidatus* Jettenia caeni” (48). An eighth MAG, nzgw510, was phylogenetically distinct from other “*Ca.* Brocadiae.” MAGs clustering with the Rifle MAG MHYJ01 (from aquifer sediment [40]) constitute clade II, which is distinct from well-known anammox bacteria (clade I) (Fig. 4a). They contained a much higher GC content (>67%) than others from this study, excluding the outlier nzgw510 (average ± SD, 42.5% ± 3%) (Table S3). A higher GC content may indicate a higher evolutionary rate for these organisms (49). All lower-GC “*Ca.* Brocadiae” MAGs from this study, including two aquifer subclades, coclustered with well-known anammox *Candidatus* genera from enrichment studies (clade I). The estimated genome sizes were significantly smaller than the high GC group (clade I, 3.22 ± 0.92 Mbp; clade II, 5.16 ± 1.24 Mbp), further suggesting a divergence in evolutionary strategy employed by the two groups.

10.1128/msystems.01255-21.4 FIG S4 Phylogenetic composition of the “*Ca.* Brocadiae” community based on 16S rRNA sequences, with oxygen concentration. (a) Stacked bar plot showing number of sequences from the rarefied OTU table containing anammox (class “*Ca.* Brocadiae”) present in samples with >4 mg/L and <4 mg/L dissolved oxygen (DO) grouped to the lowest identification level from the order “*Ca.* Brocadiales” to genus level (*, *Planctomycetes* bacterium). (b) Stacked bar plot showing the number of sequences from 37 anammox OTUs (class “*Ca.* Brocadiae”) present in samples with >4 mg/L and <4 mg/L DO. Download FIG S4, EPS file, 1.3 MB. Copyright © 2022 Mosley et al. 2022 Mosley et al. https://creativecommons.org/licenses/by/4.0/ This content is distributed under the terms of the Creative Commons Attribution 4.0 International license.

10.1128/msystems.01255-21.5 FIG S5 Genome similarity heatmap showing pairwise ANI values of 8 recovered “*Ca.* Brocadiae” genomes from this study (red) and 19 reference genomes (black). Genomes marked with asterisks are non-anammox *Planctomycetes.* Genomes in bold have >80% estimated completeness and <5% contamination. Download FIG S5, EPS file, 2.2 MB. Copyright © 2022 Mosley et al. 2022 Mosley et al. https://creativecommons.org/licenses/by/4.0/ This content is distributed under the terms of the Creative Commons Attribution 4.0 International license.

As for 16S rRNA gene analyses (Fig. 1b), “*Ca.* Brocadiae” MAG relative abundances varied across sites, corresponding to distinct geochemical conditions (Fig. 4b; Table S3) but not strictly phylogenetic relatedness (Fig. 4a). Three of four MAGs associated with well-characterized clade I anammox bacteria (nzgw511 to nzgw513) were most abundant at sites with low DO (<1.1 mg/L) and high DOC (3 to 26 g/m^3^). MAG nzgw514 is one of two closely related to “*Ca.* Brocadia.” Consistent with 16S rRNA gene data (Fig. 1b), it demonstrated a preference for oxic groundwater (Fig. 4b). Together with the high-GC MAGs (nzgw510 and clade II), it was more abundant at sites with high DO (5.6 to 7.5 mg/L) and low DOC (0 to 0.8 g/m^3^). Factors identified that influence ecological niche differentiation among anammox species include microbial growth kinetics, organic matter, oxygen tolerance, aggregation ability, and interspecific competition (5). Collectively, results here suggest that DO availability may be a major driver of anammox bacterial diversification in groundwater.

### Genomic and transcriptomic evidence for anammox metabolism and oxygen tolerance. (i) Characteristic hydrazine-based steps in anammox.

**FIG 5:**
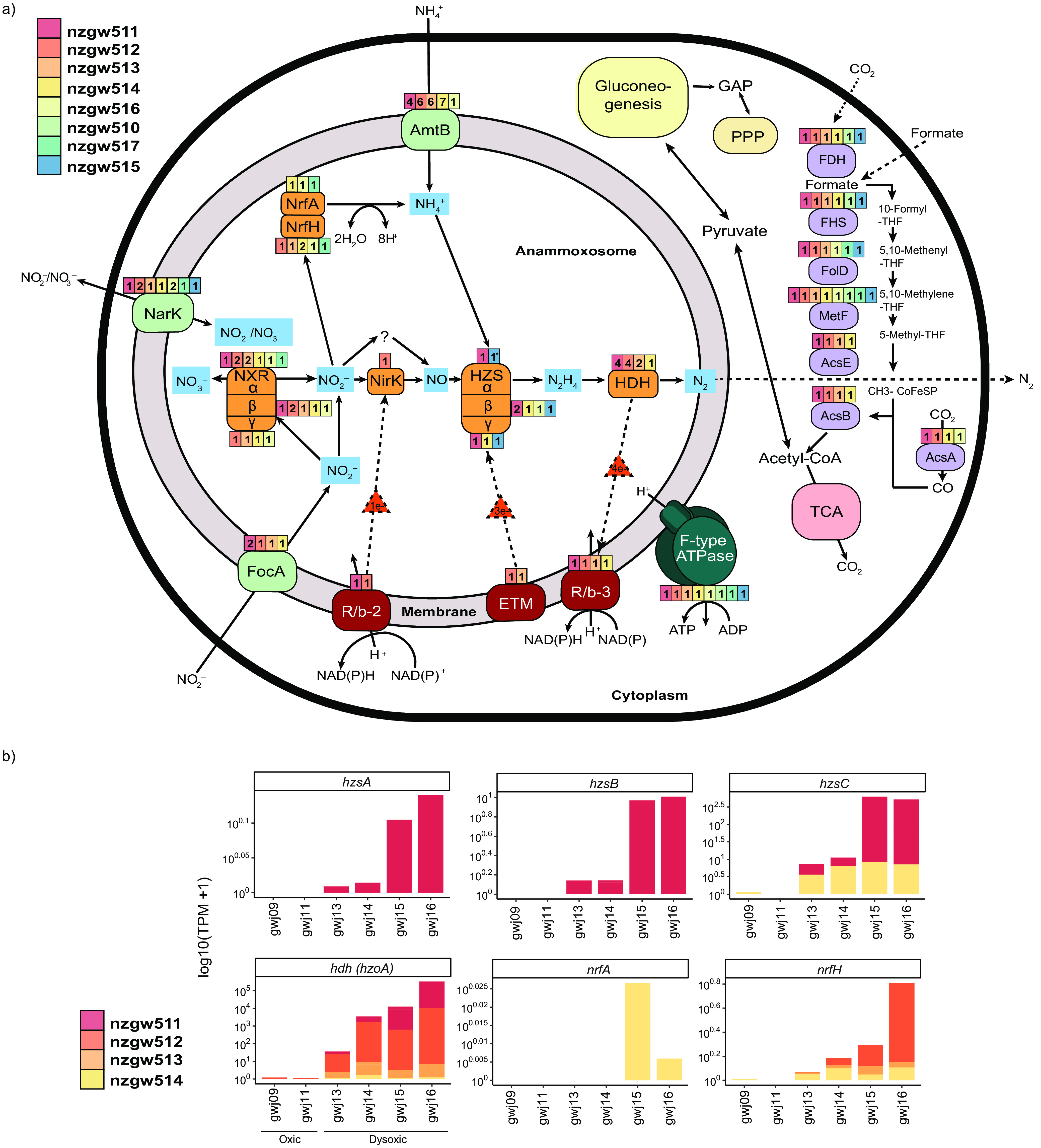
Overview of the predicted metabolic pathways of characterized anammox bacteria built from the summarized annotated data from the reconstructed genomes and mapped transcripts. (a) Metabolic pathways present in MAGs. Nxr, nitrite:nitrate oxidoreductase; Nir, nitrite reductase; Nrf, nitrite reductase forming ammonium; HZS, hydrazine synthase; HDH, hydrazine dehydrogenase; AmtB, ammonium transporters; FocA, nitrite transporters; NarK, nitrite/nitrate transporter; ETM, electron transfer module from quinone pool to HZS (composed of kuste2856 and kuste2855); R/*b*, Rieske/cytochrome *b* (*bc*_1_) complexes, R/*b*-2 (kustd1480-85), and R/*b*-3 (kuste4569-74); F-type ATPase, F-type ATP synthase (MAGs containing ≥50% of subunits); GAP, glyceraldehyde 3-phosphate; EMP, Embden-Meyerhof-Parnas pathway; FDH, formate dehydrogenase; FHS, formate–tetrahydrofolate ligase; FolD, methylenetetrahydrofolate dehydrogenase; MetF, methylenetetrahydrofolate reductase; AcsE, 5-methyltetrahydrofolate:corrinoid; AcsB, acetyl-CoA synthase; AcsA, anaerobic carbon-monoxide dehydrogenase catalytic subunit; PPP, pentose phosphate pathway; TCA, tricarboxylic acid cycle. Colors represent genomes from this study, numbers and numbers of copies present. *, partial HzsA subunit. (b) Log_10_ TPM values for active genes for hydrazine synthase, hydrazine dehydrogenase, and nitrite reductase forming ammonium from groundwater characterized as oxic (gwj09 from well SR1 and gwj11 from well SR2) and dysoxic (gwj13-14 from well E1 and gwj15-16 from well N3). Samples gwj14 and gwj16 are sonicated groundwater.

Nitric oxide produced by NO_2_^−^ reductase (50), or by cyclic feeding, is used by hydrazine synthase (HZS), along with NH_4_^+^, to produce hydrazine (N_2_H_4_) within the anammoxosome (6). Most “*Ca.* Brocadiae” MAGs recovered (6 of 8) have genes encoding signature anammox steps for hydrazine production (hydrazine synthase) and removal (hydrazine dehydrogenase) (Fig. 5; Table S4) (50). This excludes the phylogenetic outlier nzgw510 and clade II nzgw517, which have the lowest genome completeness (75% and 63%, respectively). Nonetheless, HZS genes in nzgw515 and nzgw516 indicate that clade II bacteria are capable of anammox (50) and may be important contributors to anammox in oxygen-rich groundwaters (Fig. 4b) (8). The final anammox step is catalyzed by an octaheme-hydrazine dehydrogenase (HDH [*hzoA*]). N_2_H_4_ is oxidized using cytochrome *c* as an electron acceptor (51). Although no clade II MAGs contained *hzoA*, nzgw515 contained the gene for hydroxylamine oxidoreductase, which is also capable of hydrazine oxidation *in vitro* (52). Multiple *hzoA* gene copies are present in nzgw511 to nzgw514, consistent with close phylogenetic relatedness to well-characterized anammox bacteria (Fig. 4).

10.1128/msystems.01255-21.9 TABLE S4 Functional genes present or absent based on conserved domain matches or UniProt-, BLASTP-, KEGG-, or TrEMBL-based annotations (above noise cutoff value or similarity of >30%, query coverage of >70%, E value of 0.001). Download Table S4, XLSX file, 0.02 MB. Copyright © 2022 Mosley et al. 2022 Mosley et al. https://creativecommons.org/licenses/by/4.0/ This content is distributed under the terms of the Creative Commons Attribution 4.0 International license.

**FIG 6:**
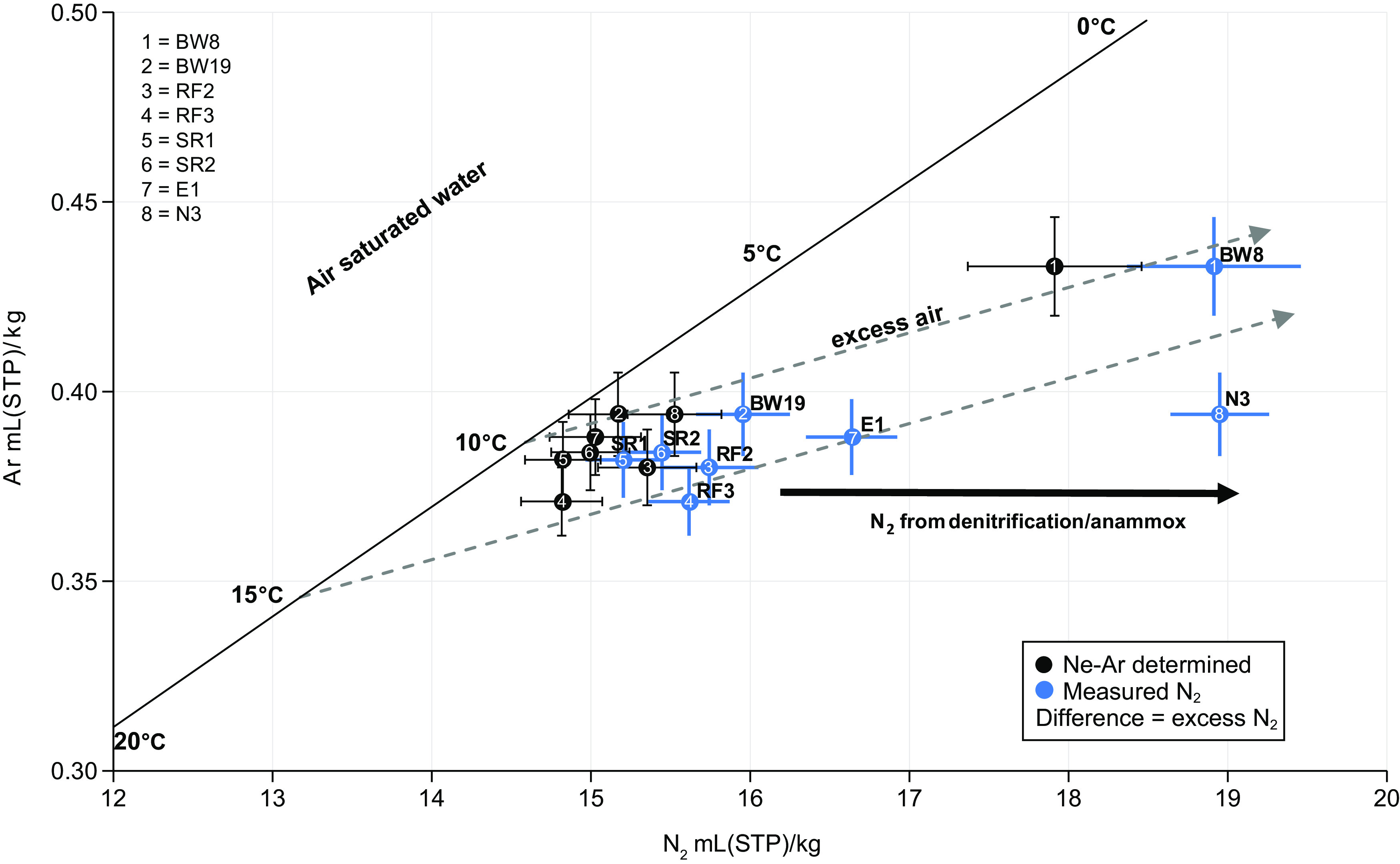
Plot of dissolved nitrogen versus dissolved argon concentrations at sites sampled for metagenomics analysis. Dissolved Ar and N_2_ are expressed in milliliters of the respective gas at standard temperature (273.15 K) and pressure (101.325 kPa) per kg of water. Bold lines represents gas concentrations in water which are in equilibrium with the atmosphere at the given temperature. Arrows indicate competing processes that can alter gas concentrations, and gray dashed lines indicate excess air in groundwater relative to atmosphere (with upper and lower lines representing addition of unfractionated excess air relative to equilibrium concentrations at 10 and 15°C). The black horizontal arrow depicts additional excess N_2_ inferred to be from biological processes (denitrification or anammox). Reconstructed N_2_ data (in equilibrium with inert atmospheric gases), based on groundwater recharge temperatures and excess air concentrations derived from dissolved Ne and Ar data (shown as black circles). Recharge temperature is the temperature of recharging water at the time it enters the groundwater system. Excess air is dissolved air in excess of the equilibrium soluble amount at the given recharge temperature (thought to originate from processes such as bubble entrapment occurring during recharge and subsequent dissolution under increased hydrostatic pressure). The difference between these and the measured N_2_ data (numbered blue circles and their shift along the *x* axis relative to the corresponding numbered black circles) indicates the amount of N_2_ in excess, formed via denitrification and/or anammox at each site. Error bars show the combined statistical standard uncertainty from all processes and calculations contributing to the measurement uncertainty, expressed as 1 standard deviation. Groundwater from wells SR1-2, BW8, BW19, and RF2-3 is characterized as oxic, while groundwater from E1 and N3 is dysoxic-suboxic (Table S1).

**FIG 7:**
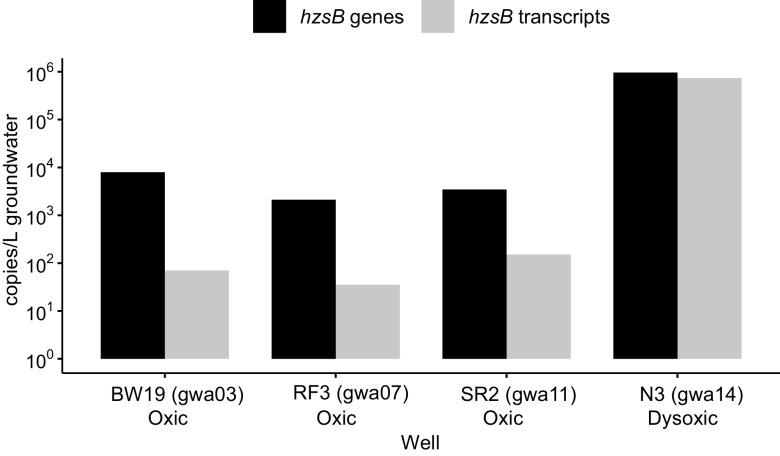
Hydrazine synthase subunit B (*hzsB*) abundance (genes and transcripts copies) from 4 wells at sites sampled for metagenomic analysis.

Reinforcing genomic evidence, metatranscriptomics data for six samples (gwj09, gwj11, and gwj13 to gwj16) across two sites revealed the transcriptional activity of *hzoA* by MAGs nzgw511 to nzgw514. This activity was 356-fold higher at the dysoxic site (wells E1 and N3) than the oxic site (Fig. 5b) and likely contributed in part to excess N_2_ measured at those sites (Fig. 6). Despite the compositional bias in anammox bacteria associated with differences in DO (Fig. 1b and 4), results confirm that in groundwater with low oxygen concentrations, anammox bacteria are more active (8). Contemporaneous measurement of excess N_2_ indicated active N_2_ generation in dysoxic groundwater (from wells E1 and N3) due to denitrification or anammox. In contrast, oxic groundwater was either at the bounds of uncertainty (wells BW8 and RF3) or devoid of measurable excess N_2_ (wells SR1-2, BW19, and RF2) (Fig. 6), which, accordingly, included groundwater with relatively little observed hydrazine synthase/dehydrogenase activity (wells SR1 and SR2). Additional quantification via ddPCR of hydrazine synthase transcripts from each site, sampled at an earlier date, further demonstrated that anammox was active in both dysoxic and oxic groundwater (Fig. 7) associated with all “*Ca.* Brocadiae” MAGs, which were distributed across eight wells over four sites (Fig. 4b; Table S3). However, quantitative results were consistent with relative metatranscriptomics data, indicating that transcriptional activity was 100-fold higher in dysoxic than oxic groundwater in the four samples analyzed.

**FIG 8:**
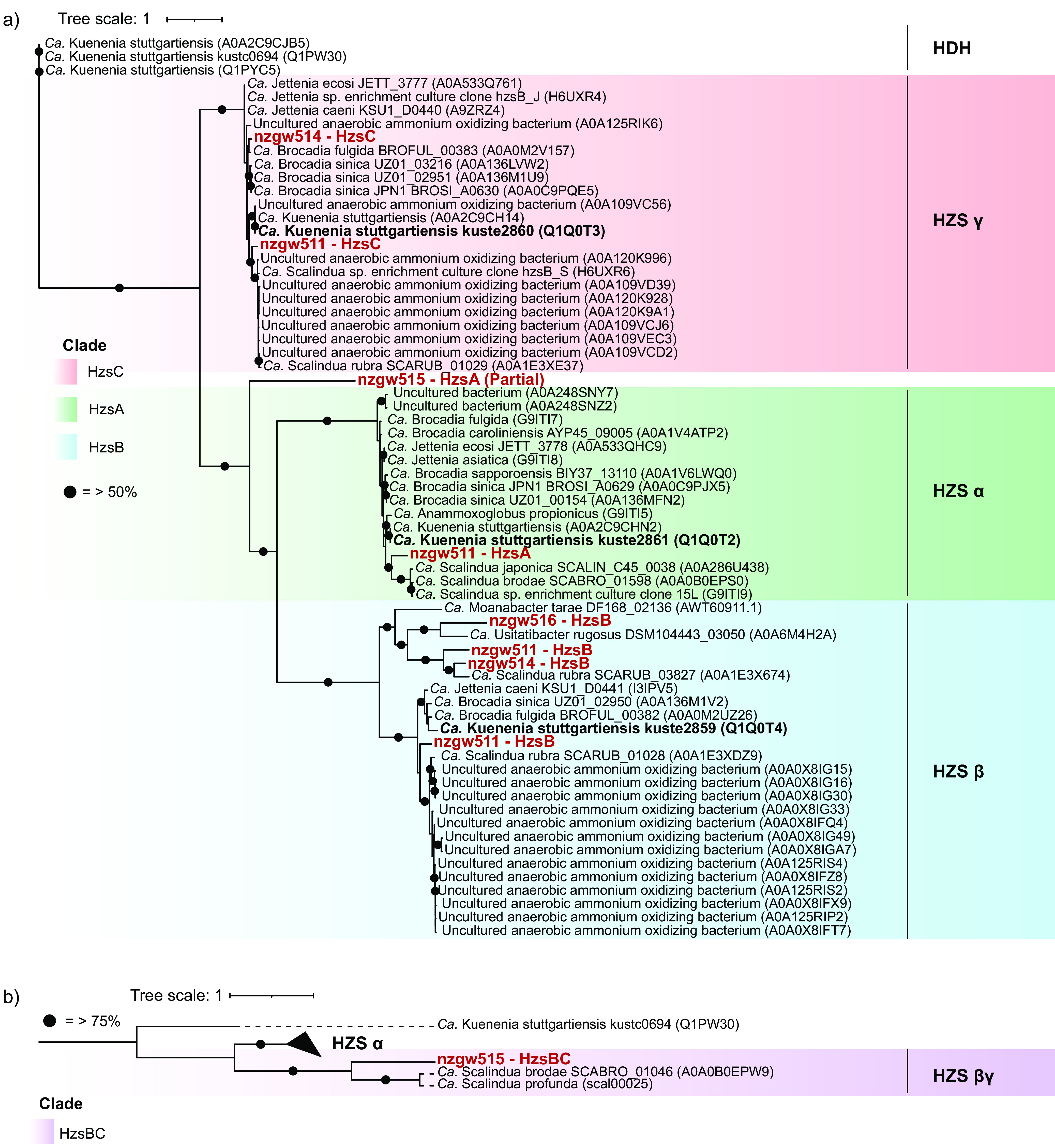
Phylogenetic tree of the recovered hydrazine synthase protein subunits from “*Ca.* Brocadiae.” (a) Phylogenetic tree with 65 protein sequences of hydrazine synthase subunits with 964 amino acid sites, built using the WAG+G4 model using 1,000 bootstraps. The hydrazine synthase (HZS) alpha, beta, and gamma proteins were predicted from protein-coding sequences recovered from genomes in the study (red) and other HZS protein sequences available from the UniProt database. HDH, hydrazine dehydrogenase. Bold black sequences are from the representative model organism “*Ca.* Kuenenia stuttgartiensis.” Circles represent >50% bootstrap values. The scale bar represents the number of substitutions per site. (b) Phylogenetic tree of 10 fused HZS beta-gamma predicted protein sequences, and another 6 HzsA (alpha subunit) sequences, made using 903 amino acid sites and built using the WAG+G4 model (1,000 bootstraps). Circles represent >75% bootstrap values; the red sequence was recovered in this study.

Further analyses showed that HZS sequence similarity and structure did not reflect “*Ca.* Brocadiae” phylogenomic relatedness. All three HZS subunits encoded by nzgw511 are closely related to “*Candidatus* Scalindua” subunits (Fig. 8). However, HzsB proteins from clade II nzgw516 and clade I nzgw514 and nzgw511 (duplicate) constitute a distinct HzsB clade relative to other anammox bacteria, indicating evolutionary divergence. Clade II nzgw515 instead encodes a fused HZS-beta-gamma protein subunit, comparable to that in the distant “*Ca.* Brocadiae” relatives “*Candidatus* Scalindua profunda” (53) and “*Candidatus* Scalindua brodae” (54).

### (ii) Production of nitric oxide (and ammonium) for anammox.

In the typical anammox reaction, the first step is NO_2_^−^ reduction to NO. To fuel anammox, the externally acquired substrates, NH_4_^+^ and NO_2_^−^, need to cross both the cytoplasmic and anammoxosome membranes (55). Multiple copies of NH_4_^+^, NO_2_^−^, and NO_3_^−^ transporters that could facilitate transport into the anammoxosome are found in all published anammox genomes (48, 53, 54, 56, 57). Five of our newly recovered genomes possess one to seven copies of *amtB*-like ammonium transporter genes (Fig. 6), which neighbor genes encoding the nitrogen-regulatory protein P-II. This protein binds directly to AmtB and regulates the ammonia channel (58). Genes encoding putative NarK NO_3_^−^/NO_2_^−^ transporters were present in seven MAGs (1 or 2 copies each). Nitrite transport is also mediated by a bidirectional NO_2_^−^/formate transporter gene, *focA* (28), which is present in four of the groundwater MAGs (1 to 4 copies each). Finally, interconversion of NO_3_^−^ and NO_2_^−^ is catalyzed by a nitrate reductase (NXR nitrate:nitrite oxidoreductase) (28). NXR genes were present in six genomes.

NO_2_^−^ reduction to NO is usually catalyzed by cytochrome *cd_1_*-type nitrite reductase (*nirS*), as found in “*Ca.* Kuenenia” and “*Ca.* Scalindua” (53, 59), or the copper-containing nitrite reductase (*nirK*), as in “*Ca.* Jettenia” spp. (60). One MAG (nzgw512) contained *nirK* similar to “*Ca.* Jettenia caeni” (48); however, other MAGs were devoid of *nirK* and *nirS* (Fig. 5). Canonical NO-forming NO_2_^−^ reductases are also lacking in several “*Ca.* Brocadia” species (e.g., “*Candidatus* Brocadia sapporoensis” and “*Ca.* Brocadia sinica”) (61) and Rifle aquifer genomes (e.g., 2-12-FULL-39-13 and 2-02-FULL-50-16-A) (40). Some anammox bacteria are proposed to utilize different enzymes to reduce NO_2_^−^ to NO (62). These enzymes may be among the hydroxylamine oxidoreductase-like octaheme proteins encoded by anammox genomes (50). Within “*Ca.* Kuenenia stuttgartiensis,” this protein (kust0457-kust0458), comprising octaheme kustc0458 and diheme kustc0457, constitutes a heterododecameric (α_6_β_6_) complex comprising 60 *c*-type hemes (50). The second is kuste4574, a close homolog of kustc0458, which forms part of a novel Rieske-heme *b* (R/*b*; *bc*_1_) complex (kuste4569 to kuste4574). It is postulated that these proteins catalyze NO_2_^−^ reduction to NO (50). Protein-coding sequences similar to kustc0457-kustc0458 and kuste4574 were identified in nzgw511, nzgw513, and nzgw514 (Table S4). These proteins may represent an alternative pathway for reducing NO_2_^−^. Furthermore, “*Ca.* Kuenenia stuttgartiensis” can grow in the absence of NO_2_^−^ by coupling ammonium oxidation to NO reduction, producing N_2_ without N_2_O emissions (63). This suggests that anammox bacteria may mitigate natural NO and N_2_O emissions in many ecosystems.

It is suggested that anammox bacteria adapt a dissimilatory nitrate reduction to ammonia (DNRA)-like metabolism in the absence of NH_4_^+^, using nitrate reductase, followed by NrfAH cytochrome *c* nitrite reductase (28). The respective products, NO_2_^−^ and NH_4_^+^, can then drive anammox. We found *nrfAH* genes in phylogenetically diverse genomes (nzgw514, nzgw516, and nzgw517). Further, genomes nzgw512 to nzgw514 contain a tetraheme cytochrome *c nrfH* gene, with CxxCH repeated sequence motifs similar to “*Ca.* Brocadia” (62) and “*Ca.* Jettenia” (60). Both *nrfA* and *nrfH* genes were observed to be expressed by these taxa in dysoxic groundwater (Fig. 5b). Together, these genes and transcripts indicate the active reduction of NO_2_^−^ to NH_4_^+^ in a NAD(P)H-dependent, six-electron transfer reaction, via a mechanism resembling DNRA (50), potentially fueling active anammox in the same sites.

NAD(P)H nitrite reductase genes (*nirD* and/or *nirB*), which also reduce NO_2_^−^ to NH_4_^+^, were found in clade II genomes, nzgw515 to nzgw517. The large subunit encodes NirB, an iron-sulfur protein. All protein-coding sequences had the highest identity (79 to 84%) to NirB from a *Planctomycetes* genome (GCA_016200125.1), recently recovered from a pristine aquifer in the United States (64). This had a geochemical profile similar to that of the site where nzgw516 and nzgw517 were most abundant (low NH_4_^+^, <0.05 mg/L, and NO_3_−, 0.23 mg/L). The *nirB* gene was recently also identified in a marine-derived anammox bacterium, related to “*Ca.* Scalindua” (65). We also identified a small subunit, NirD, in nzgw516 and nzgw517. The sequence contains a Rieske nonheme iron oxygenase family domain with closest similarity to *Verrucomicrobia* species (63.5%). NH_4_^+^ is often limiting in groundwater (20). In this study, NH_4_^+^ was undetectable in groundwater from which MAGs were recovered and was detected at only 31% of sites overall. The presence of these Nrf and Nir proteins could therefore mediate an important alternative process providing NH_4_^+^ for anammox (50).

### (iii) Mechanisms of oxygen tolerance in groundwater anammox bacteria.

All “*Ca.* Brocadiae” genomes encode proteins for oxygen protection, such as BatD and BatA, which are involved in oxygen tolerance in *Bacteroides* species (66), alkyl hydroperoxide reductases, and thioredoxin (67). Thioredoxin and peroxiredoxin genes were expressed in MAG nzgw510 at the oxic site 0.06 to 0.15 transcripts per kilobase per million reads mapped ([TPM]), and BatD, BatA, catalase, peroxiredoxin and thioredoxin were expressed by MAGs nzgw511 to nzgw514 at the dysoxic site (0.112 to 2.81 TPM). Genes encoding respiratory-chain cytochrome *c* oxidase, *cbb*_3_ type (*ccoN*, *ccoO*, and *ccoP*), were all or partially present in four MAGs (nzgw511 to nzgw514). The *cbb*_3_-type oxidase is a multichain transmembrane protein located near the anammoxosome membrane (68). This enzyme is believed to have evolved to perform a specialized function in microaerobic energy metabolism; however, it could also merely act in oxygen detoxification (28). Additionally, three clade II genomes encode a *caa*_3_-type cytochrome oxidase, containing unique CoxAB subunits. In Acidithiobacillus ferrooxidans, *coxAB* genes encode the terminal part of the ferrous iron “downhill” pathway, which shuttles electrons from extracellular iron to oxygen (69), and thus represents another potential detoxification strategy.

### (iv) Alternative metabolic pathways in groundwater anammox bacteria.

Anammox bacteria grow chemolithoautotrophically by performing CO_2_ fixation via the Wood-Ljungdahl pathway (70), and genes encoding the pathway were found in all of our groundwater genomes, including the complete pathway in nzgw511 and nzgw512 (Fig. 5; Table S4). The products of this reaction, acetyl coenzyme A (acetyl-CoA) and pyruvate, enter the oxidative tricarboxylic acid (TCA) cycle and gluconeogenesis (Fig. 5a; Table S4). However, in “*Ca.* Kuenenia stuttgartiensis,” as in four of our genomes, the oxidative branch of the TCA cycle is likely mediated by a *Re*-citrate synthase rather than a citrate synthase. MAGs nzgw511 to nzgw514 encode a *Re*-citrate synthase that was highly similar to that identified in Clostridium kluyveri (55 to 58%) (71). It operates incompletely to synthesize alpha-ketoglutarate, similar to other anaerobic bacteria (72).

In addition, several ABC transport systems were present within the genomes, revealing variations in substrate importation such as phosphate, cobalt, nickel, iron(III), zinc, sulfate, molybdate, lipoproteins, ribose, rhamnose, polysaccharides, and oligopeptides (Table S4). Oligopeptide transport systems were present in three genomes (nzgw515 to nzgw517), as seen in “*Ca.* Scalindua profunda,” and suggest these genomes are capable of oxidizing decaying organic matter (53), in addition to carbon fixation via the Wood-Ljungdahl pathway (Fig. 5a). Additionally, iron(III) transporters were present in three genomes, which may enable the coupling of formate oxidation to iron(III) reduction in the absence of NO_2_^−^ or NO, as previously identified in other species (73). Hydrogenase genes were also present in three of the genomes. The first encodes a [Ni-Fe] group 3b hydrogenase, found in nzgw511, which is proposed to harbor (sulf)hydrogenase activity (74). This genome also encoded a sulfate adenylyltransferase, which may play a role in sulfide oxidation (75). The second is a group 4 hydrogenase present in nzgw513. These hydrogenases carry out coupled oxidation of NADPH to the evolution of H_2_ (76), indicating hydrogen turnover and/or an alternative energy source. Our results therefore suggest these groundwater anammox bacteria are metabolically versatile, containing various hydrogenases and ABC transporters for organic compounds and genes encoding DNRA, which would allow for growth under substrate-limiting conditions (such as low ammonium concentrations).

### Implications for groundwater.

Findings presented here indicate that natural and agriculturally derived nitrate and ammonium (77) may be removed from aquifers with a range of bulk oxygen concentrations by endogenous anammox bacteria. In this study, gene transcripts indicative of anammox were positively correlated with those for aerobic ammonia oxidation, which could fuel the anammox process by providing nitrite, across anoxic-to-oxic conditions. These two processes are considered to have opposing oxygen requirements; however, some oxygen in groundwater may facilitate loss of fixed N by stimulating aerobic ammonia oxidation. Additionally, although anammox bacteria have been shown to outcompete denitrifiers in low-carbon groundwaters (8), we found no evidence that increased DOC availability negatively impacted anammox bacteria (overall) or anammox-related activity. While DOC is required by heterotrophic denitrifying bacteria (1), its influence on denitrifiers (autotrophic or heterotrophic) and anammox bacteria is likely complex. For instance, DOC plays a role in the regeneration of ammonium (78), essential for anammox, and oxygen depletion stimulated by DOC creates conditions favorable for both competing pathways leading to N_2_. Moreover, genomic data indicate that anammox bacteria in groundwater may be capable of supplementing their metabolism through the acquisition of exogenous organic carbon. Further studies are needed to validate the oxygen tolerances and metabolic versatility of anammox bacteria in the terrestrial subsurface.

### Conclusions.

This study shows that anammox bacteria are prevalent and active across wide-ranging aquifer chemistries and lithologies, including in oxic groundwater, although this is predicted to be unfavorable. While we found significantly more “*Ca.* Brocadiae” diversity in anoxic groundwater, some taxa were positively associated with DO concentrations. Of the eight novel “*Ca.* Brocadiae” genomes reconstructed, three belong to undercharacterized clades previously recovered only from aquifers. Most possess genes involved in signature pathways of anammox, including novel hydrazine synthase genes. A co-occurrence of anaerobic and aerobic ammonia oxidizers at many sites suggests metabolic handoffs (such as nitrite) between these processes. Furthermore, genomic characterization of “*Ca.* Brocadiae” identified a range of potential aero-tolerance mechanisms, explaining our finding of anammox in oxic groundwaters. Results indicate that niche differentiation occurs among anammox bacteria based on oxygen concentrations and that anammox is a common mechanism for nitrogen removal in aquifers.

## MATERIALS AND METHODS

### Study sites and sample collection.

Groundwater was collected from 59 wells spanning 10 aquifers in the Waikato, Wellington, and Canterbury regions of New Zealand (Table S1), from several aquifer lithologies: alluvial sandy gravel (71 samples), sand-silt (1 sample), basalt (1 sample), shell bed (1 sample), peat (1 sample) and ignimbrite (6 samples). Wells were purged (∼3 to 5 bore volumes); then, 3 to 90 L of groundwater or 0.5 to 15 L of biomass-enriched groundwater was filtered on site. Biomass-enriched groundwater was collected from 8 sites in Canterbury (Table S1) directly following standard groundwater collection, and low-frequency sonication (2.43 kW) for 2 min to detach biofilms and aquifer particles (also covered in biofilms) (79). Biomass was captured onto mixed cellulose ester membrane filters (1.2-μm-pore-size prefilter over a 0.22-μm filter), using a 14-mm stainless steel filter holder (Merck Millipore Ltd., Cork, Ireland). Both filters were immediately submerged in RNA Later (Thermo Fisher Scientific, Waltham, MA, USA), transported on dry ice, and stored at −80°C.

### Chemical analysis of water samples.

Dissolved oxygen (DO), water temperature, pH, oxidation-reduction potential (ORP), and specific conductance (SPC or conductivity) were measured on-site using a flowthrough cell and field probes (YSI EXO sonde 2, YSI PRO+, and YSI ProDSS; YSI, Yellow Springs, OH, USA). Samples were categorized by DO concentration as follows: anoxic (0 mg/L), suboxic (<0.3 mg/L), dysoxic (0.3 to 3 mg/L), and oxic (>3 mg/L) (33). Unfiltered groundwater was analyzed for P, N, C, S, Mn, and alkalinity at Hill Laboratories (Hamilton, New Zealand). Total phosphorus and phosphate were determined according to American Public Health Association method 4500-P (APHA 4500-P), parts B and E, (modified to include an acidic ammonium persulfate to convert organophosphates and polyphosphates to orthophosphate) (80). Dissolved reactive phosphorus (DRP) was determined according to APHA 4500-P, part G (sample was reacted with ammonium molybdate and ascorbic acid to form molybdenum blue and then detected at 880 nm). Total ammoniacal N was determined according to APHA 4500-NH3, part H (using a phenol/hypochlorite reaction forming a complex that was detected at 630 nm), and calculated as NH_4_-N = NH_4_^+^-N + NH_3_-N. Nitrite N and nitrate N + nitrite N were determined according to APHA 4500-NO_3_, part I (NO*_x_* N is measured via automated cadmium reduction and Griess reaction; NO_2_-N is calculated by automated Griess reaction [sulfanilamide] detected at 540 nm). Nitrate N was calculated as (nitrate N + nitrite N) − nitrite N. Total organic carbon (TOC) and dissolved organic carbon (DOC) were measured according to APHA 5310, part C (analyzed using supercritical persulfate oxidation with phosphoric acid and sodium persulfate), where TOC is calculated as total carbon − total inorganic carbon. For DOC, groundwater was filtered first using a 0.45-μm polypropylene filter. Total suspended solids were measured by first evaporating groundwater samples in an oven at 105°C until dry. Dried solids were then weighed and normalized to the total water volume analyzed. Sulfate was measured according to APHA 4110 B. Alkalinity and dissolved manganese were analyzed according to APHA 2320 B and APHA 3125 B, respectively.

To assess denitrification/anammox, excess N_2_ gas was quantified using methods by Martindale et al. (81). Briefly, dissolved Ar, Ne, and N_2_ were measured by a standard curve via a system comprising two detectors, a pulsed-discharge helium ionization detector (Valco Instruments D-4-I-SH14-R), a thermal conductivity detector (Shimadzu TCD-2014), and ultrahigh-purity helium (He) gas. The measurement of a sample uses the principles of headspace analysis and Boyle’s law (81). Excess N_2_, attributable to denitrification/anammox, was determined by comparison to inert Ar and Ne gas concentrations. The uncertainty was reported for each measurement of the original sample concentration as the standard measurement error (combined standard uncertainty).

### Nucleic acid extraction and sequencing. (i) Nucleic acid extraction.

RNA and DNA were extracted using RNeasy PowerSoil Total RNA and DNeasy PowerSoil Pro kits (Qiagen, Valencia, CA, USA), respectively, with nuclease-free glycogen added to aid RNA precipitation (0.1-μg/μL final concentration; Roche Diagnostics, Basel, Switzerland). DNA extractions used 0.14 to 0.89 g of crushed filter (1 to 47 extractions per sample). For RNA, 2.12 to 3.90 g was used per extraction (1 extraction per sample). RNA was DNase treated using the Turbo DNA-free kit (“rigorous” protocol; Invitrogen, Carlsbad, CA, USA). DNA removal was verified via 16S rRNA gene amplification (as described below, but over 55 cycles) and gel electrophoresis. The products of replicate DNA extractions were pooled and concentrated using sodium acetate (0.3 M final concentration) and ethanol (2× volume) precipitation with 0.1 μg/μL glycogen (Roche Diagnostics) via ethanol precipitation. RNA extractions were concentrated using the Zymo RNA Clean and Concentrator-5 kit (Zymo Research, Irvine, CA, USA).

High-molecular-weight DNA for whole-genome shotgun (WGS) sequencing was verified via 1% agarose gel electrophoresis. Nucleic acids were quantified with a Qubit 3.0 fluorometer (Thermo Fisher Scientific, Waltham, MA, USA) using double-strand DNA (dsDNA) HS and RNA HS assay kits and quality checked using a Nanophotometer (Implen, Munich, Germany). RNA was checked using an Agilent Bioanalyzer with RNA 6000 Nano and Pico chips (Integrated Sciences, NSW, Australia). Samples with an RNA integrity number of 6 or more or a fragment distribution value (DV200) of >30% (percentage of RNA fragments above 200 nucleotides) were used to quantify transcripts and for transcriptomics. Between 24 pg and 1.1 μg of total RNA was converted to cDNA using Superscript III Supermix (Invitrogen, Carlsbad, CA, USA).

### (ii) Metagenome, metatranscriptome, and amplicon sequencing.

DNA libraries for 15 samples (gwj01 to gwj16) were prepared using the TruSeq Nano DNA kit (Illumina, San Diego, CA, USA), with a targeted insert size of 550 bp, at the Otago Genomics Facility (University of Otago, New Zealand), except low-yield sample gwj02, which was prepared with the ThruPLEX DNA-seq kit (TaKaRa Bio USA, Inc., Mountain View, CA, USA). Then, 2 × 250 bp sequencing was performed using the Illumina HiSeq 2500 V4 platform. RNA libraries were prepared using the Ovation SoLo RNA-Seq system (NuGEN, Redwood City, CA, USA) using custom probes for rRNA depletion at the Otago Genomics Facility. Custom rRNA probes were designed by the manufacturer using small and large ribosomal subunit sequences reconstructed from the 16 metagenomes with EMIRGE (82) over 40 iterations with clustering at 97% identity and using the SILVA 132 database (83). Ribosomal sequences generated were used as target sequences in the design of custom AnyDeplete probes using NuGEN’s proprietary algorithm. rRNA and genomic DNA were removed as a part of the library preparation kit, using the custom probes and DNase treatment, respectively. Paired-end 2 × 125 bp reads were generated from RNA libraries using the Illumina HiSeq 2500 V4 platform.

PCR amplification of 16S rRNA genes used modified Earth Microbiome Project primers EMP-16S-515′F and EMP-16S-806′R primers (84, 85) with Illumina Nextera adapters and MyTaq HS Red mix (Bioline, London, UK). PCR cycling conditions were as follows: 95°C for 5 min, 35 cycles consisting of 45 s at 95°C, 60 s at 50°C, and 90 s for 72°C, followed by 72°C for 10 min. Amplicons were purified using Agencourt AMPure XP beads (Beckman Coulter, Brea, CA, USA). Barcoded libraries, prepared according to Illumina's 16S metagenomic sequencing library preparation manual, were loaded with 10% PhiX for 2 × 250 bp sequencing via Illumina MiSeq with V2 chemistry (Auckland Genomics, University of Auckland, Auckland, New Zealand).

### Amplicon processing.

Sequences were quality checked using FastQC v0.11.7 (86), and merged using USEARCH v9.0.2132 (87). Sequences were quality filtered using sickle (minimum Phred score, ≥30; length, ≥200 bp) with another 10 bp of lower-quality sequence removed from each end using USEARCH -fastx_truncate (87). Sequences were dereplicated and clustered at 97% similarity with chimera removal to generate operational taxonomic units (OTUs) using the UCLUST pipeline (88). OTUs were classified using USEARCH -sintax with the SILVA SSU Ref NR99 database v132 (83). Nonprokaryotic and singleton sequences were removed before rarefying to 13,393 using QIIME2 v2018.2 (89). For comparison, amplicon sequence variants were also generated by methods described previously (90), and with a trimmed merged read length of 251 bp.

### Quantitative PCR of hydrazine synthase and ammonia monooxygenase genes.

Droplet digital PCR (ddPCR) via the Bio-Rad QX200 platform used 20-μL reactions with 10 μL 2× QX200 ddPCR EvaGreen Supermix (Bio-Rad, Hercules, CA, US), 0.4 μM forward and reverse primers, 1 μL of DNA (<0.2 to 10.4 ng/μL) or cDNA (<0.2 to 5.1 ng/μL), and nuclease-free water. Primers for hydrazine synthase (hzsB_396F and hzsB_742R) (91) were used with the following cycling conditions: 95°C for 10 min and then 40 cycles consisting of 60 s at 95°C, 60 s at 59°C, and 45 s at 72°C, followed by 72°C for 15 min, 4°C for 5 min and 90°C for 5 min and holding at 12°C. Primers for archaeal ammonia monooxygenase (Arch-amoAF and Arch-amoAR) (92) were used with the following conditions: 95°C for 5 min, then 40 cycles consisting of 95°C for 45 s, 53°C for 60 s, and 72°C for 90 s, followed by 72°C for 15 min, 4°C for 5 min, and 90°C for 5 min and holding at 12°C. The bacterial *amoA* primer pair amoA1F and amoA-modR (93, 94) was also used with the following conditions; 95°C for 5 min, then 40 cycles consisting 95°C for 30 s, 40 s at 55.8°C, and 40 s at 72°C, followed by 72°C for 2 min, 4°C for 5 min, and 90°C for 5 min and holding at 12°C. Negative controls used diethyl pyrocarbonate (DEPC)-treated water. Positive controls used gBlocks dsDNA fragments of *hzsB* (clone-hzsB-MP3-17ss-02-hzsB; GenBank accession no. KP002830.1) and *amoA* genes (gi409108657-arch-amoA [GenBank accession no. JX488453.1] and ATCC 25196-bact_amoA [GenBank accession no. CP000103.1]) (Integrated DNA Technologies, Coralville, IA, USA). Data were analyzed using the QuantaSoft software package (Bio-Rad). Threshold values for positive droplets were set based on the amplitude of negative and positive droplets using the positive control as a reference.

### Metagenome assembly and genome binning.

Adapters were removed from metagenomic reads using Cutadapt (95). Reads were trimmed with sickle (Phred score ≥ 30; length ≥ 80 bp) and quality checked using FastQC v0.11.7 (86). All samples were individually assembled using SPAdes v3.11.1 (96) (–meta, -k 43,55,77,99,121). Coassemblies were performed on samples from the same site and sample type (groundwater or groundwater plus attached fraction) using identical parameters. Scaffolds of ≥2 kb were binned with MetaBAT2 v2.12 (97), MaxBin v2.2.6 (98), and CONCOCT v1.0.0 (99). The best-scoring bins per assembly were selected with DAS_Tool v1.1.1 (100). Bins were dereplicated across assemblies using dRep v2.0.5 (ANI > 99%; completeness > 50%) (101) and manually refined using t-SNE (t-distributed stochastic neighbor embedding) transformation of tetranucleotide frequencies and coverage (https://github.com/dwwaite/bin_detangling). Genome completeness was estimated using CheckM (102). For genome coverage, trimmed reads were mapped onto dereplicated genomes using bowtie2 v2.3.2 (103) (-n 1 -l 222 –minins 200 –maxins 800 –best). Sample-specific genome relative abundance was calculated by normalizing to library size and highest read count (104). Estimated genome size was calculated as [bin size − (bin size × contamination)]/completeness (105).

### Metabolic predictions.

Protein-coding gene sequences were predicted using Prodigal v2.6.3 (106) and annotated using USEARCH v9.02132 (87) with -usearch_global (–id 0.5 –evalue 0.001 –maxhits 10) and the UniRef100, UniProt (107), and KEGG databases (108). Hidden Markov model (HMM) searches were carried out using HMMER v3.3 (109) against the PFAM (110) and TIGRfam (111) databases and databases from Anantharaman et al. (40) (for HMM individual cutoffs, see Table S5). BLASTP (National Center for Biotechnology Information [NCBI]) was used to identify predicted protein-coding sequences for Rieske/cytochrome *b* systems and the electron transfer module from “*Ca.* Kuenenia stuttgartiensis” (similarity > 30%; query coverage > 70%) (Tables S4 and S5).

10.1128/msystems.01255-21.10 TABLE S5 Databases used for functional gene search parameters (Pfam, TIGRfam, and KEGG) with respective cutoff scores (Pfam and TIGRfam). BLASTP (NCBI nr database) and KEGG matches (similarity >30, query cover >70%, E value = 0.001). Download Table S5, XLSX file, 0.03 MB. Copyright © 2022 Mosley et al. 2022 Mosley et al. https://creativecommons.org/licenses/by/4.0/ This content is distributed under the terms of the Creative Commons Attribution 4.0 International license.

### Genome classification and reconstruction/recovery of 16S rRNA gene sequences.

Metagenome-assembled genomes (MAGs) were taxonomically classified using the Genome Taxonomy Database taxonomic classification tool, GTDB-Tk v0.2.1 (112). 16S rRNA gene sequences were reconstructed from metagenomic data using EMIRGE (82) or SPAdes (96) with identification by Metaxa2 (113) and PATRIC (114). EMIRGE was used to reconstruct 16S rRNA gene sequences from metagenomes with 97% cluster identity, 80 iterations, and the SILVA SSU Ref NR99 database v132 (83).

### Phylogenetic and protein sequence trees.

Bacterial core gene alignments (75 to 114 genes per genome) generated from GTDB-Tk were used to construct a maximum-likelihood phylogenomic tree in IQ-TREE (v1.6.9) (115) using ModelFinder (116) best-fit model LG+F+R5, and annotated with iTOL (117). EMIRGE, Metaxa2, and amplicon sequences were aligned using MUSCLE (118) with default parameters and trimmed to 295 bp using Geneious 11.1.2. A maximum-likelihood tree was constructed as with IQ-TREE using ModelFinder best-fit model TIM3+F+I+G4. Protein sequences of hydrazine synthase subunits A, B, and C were aligned with MUSCLE and trimmed to remove columns with >50% gaps using trimAl (119). A maximum-likelihood tree was constructed as with IQ-TREE using ModelFinder best-fit model WAG+G4.

### Metatranscriptome processing.

Adapters were removed from transcriptomic reads and then quality trimmed and checked as described above for metagenomic reads. Residual rRNA sequences were removed using SortMeRNA v2.1 (120). Additional checks were carried out to ensure that filtered reads were still paired, and read pairs were ordered using a repair script from BBMap v38.81 (121). Filtered transcriptomic reads were mapped to contigs from the set of dereplicated MAGs using Bowtie2 (103) (v2.3.5, –end-to-end –very_sensitive). Read counts were determined using featureCounts (122) (v1.6.3, -F SAF). Singleton reads per gene were removed, and the remaining read counts were normalized to transcripts per kilobase per million reads mapped (TPM) using the following equation: (number of reads mapped to gene) × (1,000/gene length) × (1,000,000/library size) (123).

### Statistical analyses.

Analyses were carried out in RStudio (v4.0.3) (124) with the packages vegan v2.5.6 (125) (for distance-based redundancy analysis [dbRDA]) and phyloseq v1.34.0 (126) (alpha diversity analysis). All correlations were Spearman’s rank correlations (a *P* value of <0.05 was considered significant) and adjusted using the Benjamini-Hochberg (BH) method. To determine differentially abundant taxa between sample groups, linear discriminant analysis effect size (LEfSe) was performed on rarefied OTUs using the Galaxy computational tool (http://huttenhower.sph.harvard.edu/galaxy/).

### Data availability.

All metagenomic, metatranscriptomic, and amplicon sequence data are available from NCBI under BioProject no. PRJNA699054.
