# Supplementary figures and images for: Metabolic Diversity and Aero-Tolerance in Anammox Bacteria from Geochemically Distinct Aquifers
Source: mSystems. 2022 Feb 22;7(1):e01255-21. doi: 10.1128/msystems.01255-21 (PMC8862662; doi:10.1128/msystems.01255-21)

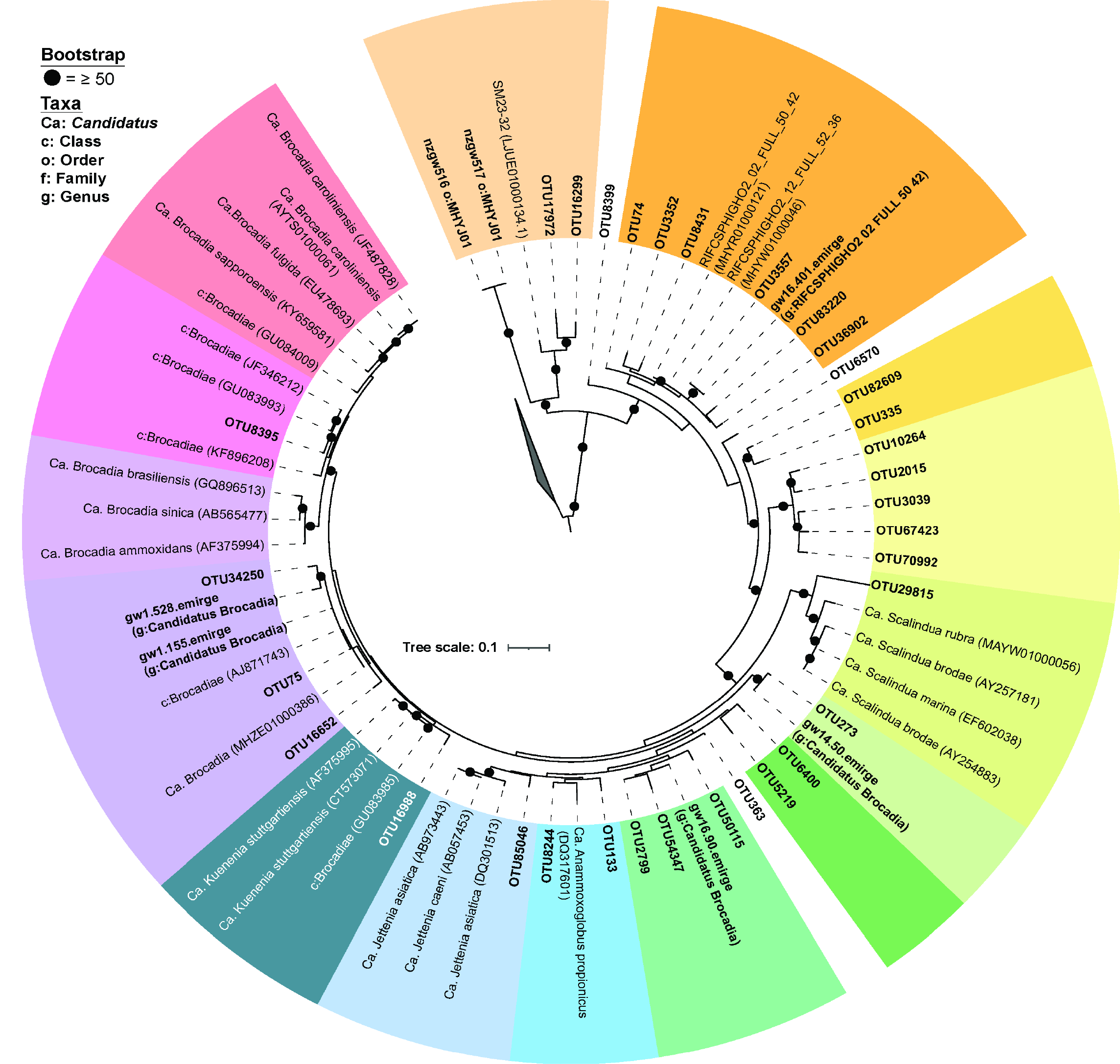

Supplement: FIG S1 [file msystems.01255-21-sf001.tif]
